# High-Speed GPU-Based Fully Three-Dimensional Diffuse Optical Tomographic System

**DOI:** 10.1155/2014/376456

**Published:** 2014-04-10

**Authors:** Manob Jyoti Saikia, Rajan Kanhirodan, Ram Mohan Vasu

**Affiliations:** ^1^Department of Physics, Indian Institute of Science, Bangalore 560012, India; ^2^Department of Instrumentation and Applied Physics, Indian Institute of Science, Bangalore 560012, India

## Abstract

We have developed a graphics processor unit (GPU-) based high-speed fully 3D system for diffuse optical tomography (DOT). The reduction in execution time of 3D DOT algorithm, a severely ill-posed problem, is made possible through the use of (1) an algorithmic improvement that uses Broyden approach for updating the Jacobian matrix and thereby updating the parameter matrix and (2) the multinode multithreaded GPU and CUDA (Compute Unified Device Architecture) software architecture. Two different GPU implementations of DOT programs are developed in this study: (1) conventional C language program augmented by GPU CUDA and CULA routines (C GPU), (2) MATLAB program supported by MATLAB parallel computing toolkit for GPU (MATLAB GPU). The computation time of the algorithm on host CPU and the GPU system is presented for C and Matlab implementations. The forward computation uses finite element method (FEM) and the problem domain is discretized into 14610, 30823, and 66514 tetrahedral elements. The reconstruction time, so achieved for one iteration of the DOT reconstruction for 14610 elements, is 0.52 seconds for a C based GPU program for 2-plane measurements. The corresponding MATLAB based GPU program took 0.86 seconds. The maximum number of reconstructed frames so achieved is 2 frames per second.

## 1. Introduction

Diffuse optical tomography using low energy near infrared light (NIR) is relatively inexpensive modality for in vivo and noninvasive functional imaging of soft tissue up to depths of several centimeters. DOT provides valuable functional information through the recovery of spectral variation of the optical absorption [*μ*_*a*_; *λ*] and scattering coefficient [*μ*_*s*_; *λ*] [1–6]. The concentration of hemoglobin, lipids, and water [7] is very valuable information from the diagnostic point of view. The DOT also has unique functional brain imaging capabilities [8–10]. The advantages include functional near-infrared spectroscopy (fNIRS), portability, and comprehensive hemodynamic measurement [8–10]. Since breast is a soft tissue, early breast cancer detection has been the primary application of DOT [6, 11]. The DOT system uses NIR light source (laser diode or LED) to illuminate different position of the tissue surface and light detectors measure the transmitted light at specific surface positions. The parameter recovery known as inverse problem in highly scattering biological tissues is a nonlinear and ill-posed problem and is generally solved through iterative methods. The iterative algorithm uses a forward model to arrive at a flux density (computed flux density based on the initial absorption and scattering coefficients) at the tissue boundary. The forward model uses light transport models such as stochastic Monte Carlo simulation [12] or deterministic methods such as radiative transfer equation (RTE) [13] or a simplified version of RTE, namely, the diffusion equation (DE) [14]. The RTE is the most appropriate forward model for light transport through an inhomogeneous material [3, 4, 15–17]. The RTE has many advantages which include the possibility of modelling the light transport through an irregular tissue medium. The exact solutions for the RTE exist only for simple cases such as isotropic scattering in simple geometries [18]. Therefore one needs to make further approximations or compute numerical solutions. By expanding the RTE in spherical harmonics, one can derive a hierarchy of equations [19, 20], of which the simplest, the so-called P1 approximation, is the diffusion equation. The diffusion equation is generally used for computer implementations using finite element based discrete models. Gauss-Newton method [2] is used for solving the DOT problem. The diffusion equation is valid under the condition that absorption coefficient [*μ*_*a*_; *λ*] is much smaller than scattering coefficient [*μ*_*s*_; *λ*]. The numerical methods used for discretizing the diffusion equation are the finite difference method (FDM) [1], boundary element method (BEM) [21, 22], and the finite element method (FEM) [2]. The FEM, which considers that the solution region comprises many small interconnected tiny subregions, gives a piecewise approximation to the governing equation; that is, the complex partial differential equation is reduced to a set of linear or nonlinear simultaneous equations. Thus the reconstruction problem is a nonlinear optimization problem where the objective function defined as the norm of the difference between the model predicted flux and the actual measurement data for a given set of optical parameters is minimized. One method of overcoming the ill-posedness is to incorporate a regularization parameter. Regularization methods replace the original ill-posed problem to a better conditioned but related one in order to diminish the effects of noise in data and produce a regularized solution to the original problem.

In the Broyden based approach, with an initial uniformly distributed optical parameter (*μ* ^0^ = *μ* ^guess^) Jacobian is calculated only once and thereafter, in each iteration, Jacobian is updated using rank-1 update. The forward model is solved for model predicted flux [*M* ^*C*^ = *F*(*μ* ^*i*^)]. The difference between predicted measurement and the experimental measurement [Δ*M* = *M* ^*E*^ − *M* ^*C*^] is used for updating the Jacobian [*J*_*i*+1_ = *J*_*i*_ + Δ*J*_*i*_] [23]. In a conventional approach, the computation time for Jacobian estimation takes a good portion of the reconstruction time. With the adaptation of Broyden method, the computation time for Jacobian update has been brought down by an order of magnitude. However, the computing time for a 3D reconstruction is still an impediment for functional imaging. The number of frames per second achievable is still very low. To overcome this challenge, the tremendous computational power of multithreaded GPU is employed to perform parallel computation. GPU is adopted for scientific simulation over other alternative parallel processors such as cluster of workstations due to its affordability, portability, and computation power in terms of Giga-floating point operations per second (GFLOPS) and user friendly parallel programming platform CUDA [24]. Of late, the availability of parallel programming support for GPUs provided by MATLAB [25] provides a much simpler interface for utilizing the enormous computing power of GPUs.

Researchers have started using GPU and CUDA technology in recent time for solving a large number of applications. These include problems associated with tomography such as iterative algebraic reconstruction (ART), a 3D convolution back-projection algorithm for X-ray tomography [26], multiscale image fusion algorithm [27], and the solution of many engineering and scientific problems by Jacobi's iterative approach [28]. A fast Monte Carlo simulation of ultrasound-modulated light using a GPU has been reported by Leung and Powell [29]. The GPU-based parallel Monte Carlo algorithm has been developed by Alerstam et al. [30]. Prakash et al. [31] used a CUDA enabled GPU for the implementation of 3D DOT reconstruction algorithm. They evaluated the performance of CULA (CULA is a set of GPU-accelerated linear algebra libraries utilizing the CUDA parallel computing architecture) [32] based algorithms for DOT. Schweiger [33] studied a GPU-accelerated finite element method for modelling light transport in diffuse optical tomography. A significant performance improvement (5 to 30) was obtained when they parallelized the DOT program based on TOAST [34] using GPUs. Freiberger et al. [35] developed a scheme to implement fluorescence tomography on GPU hardware and a performance improvement of 15 was reported.

In this study, we have developed a GPU-based high-speed (at least 2 frames per second reconstruction) fully 3D tomographic system for diffuse optical tomography. One of the most computationally expensive components of the iterative DOT algorithm, the reevaluation of the Jacobian in each of the iterations, is avoided by using the Broyden update formula that provides a rank-1 update to the Jacobian. The second factor that aids in bringing down the execution time is the availability of multinode (2496 nodes) multithreaded (with limit being 65536 threads) GPUs having a large number of cores and CUDA software architecture. The focus is on development of a GPU implementation of a direct 3D DOT reconstruction algorithm to boost the computation speed. The basic requirement for a medical diagnostic equipment is that a physician should be able to view the reconstructed images as the patient is undergoing scan. The functional imaging calls for at least 5–10 frames per second reconstruction. The reconstruction time for a 3D image normally takes more time (few hours) and so reconstructions are mostly carried out as offline operations. In our implementation, the forward computation uses finite element method (FEM) and the problem domain is discretized into 14610, 30823, and 66514 tetrahedral elements, respectively, for a cylindrical object of 60 mm diameter and 70 mm height. The reconstruction time, so achieved for one iteration of the DOT reconstruction for 14610 elements, is 0.78 seconds for a C GPU system for 3 planes measurements. The corresponding GPU-MATLAB program took 1.29 seconds. For a 2-plane measurement system, the corresponding reconstruction times are 0.52 and 0.86 seconds for C-CUDA and GPU-MATLAB, respectively. The maximum number of reconstructed frames so achieved is 2 frames per second.

Two different GPU implementations of DOT programs are developed in this study: (1) one uses conventional C language program augmented by CULA-based GPU routines. (2) The second one is based on MATLAB development tools supported by MATLAB parallel computing tools for GPU. The computation times of the algorithm on host CPU and GPU configurations are presented for C, C-CUDA/CULA, and MATLAB implementations. An analysis of the execution profile gives the time utilization of the host CPU and GPU while running various tasks of the reconstruction algorithm, which allows us to identify the tasks that need a closer watch for optimization. The DOT algorithm is an inverse problem that requires an iterative solution and uses a forward model based measurement data estimator and an inverse computation path that updates the absorption and scattering coefficient map. The algorithms are evaluated by making use of the mean square error, both for simulated and experimental data. The mean square error of *μ*_*a*_^estimated^ − *μ*_*a*_^actual^ is also plotted for simulation results.

## 2. Methodology

### 2.1. Newton-Based MoBIIR (Model Based Iterative Image Reconstruction) Approach

The time independent diffusion equation (DE) for the light transport *F*(*μ*_*a*_, *κ*) is given as [1–6]

(1) $$\begin{array}{c} -\nabla \cdot \kappa \left(r\right)\nabla \Phi \left(r,t\right)+\mu_{a}\left(r\right)\Phi \left(r,t\right)=q_{0}\left(r,t\right), \end{array}$$

where photon density $\Phi (r,t)=\int_{4\pi }I(r,t,{\hat{e}}_{s})d^{2}{\hat{e}}_{s}$, energy radiance is $I(r,t,{\hat{e}}_{s})$ at position *r* into direction ${\hat{e}}_{s}$, diffusion coefficient *κ*(*r*) = 1/3[*μ*_*a*_(*r*) + *μ*_*s*_′(*r*)], and *μ*_*a*_ and *μ*_*s*_′ are absorption coefficient and reduced scattering coefficient, respectively. The diffusion equation is valid only when *μ*_*s*_′ ≫ *μ*_*a*_, which is true for most of the biological tissues in the near-infrared region. The input photon is from a source of constant intensity (*A*_*dc*_), modulated by a sinusoidal current of amplitude *A*_*ac*_ and frequency *ω*_0_ located at *r* = *r*_0_. The transmitted output optical signal is of the form *A*_*dc*_ + *A*_*ac*_cos⁡(*ω*_0_ *t* + *ϕ*) and we measure amplitude and phase by lock-in detection method.

The boundary condition (Robin boundary condition) is given by

(2) $$\begin{array}{c} 2Ak\left(r\right)\frac{\partial \Phi \left(r\right)}{\partial n}+\phi \left(r\right)=0\;\forall r\in \Omega , \end{array}$$

where the term A is Fresnel reflection coefficient at the boundary.

The DOT problem is represented by a nonlinear operator given by *F*(*μ*_*a*_, *κ*). *M* is the measurement vector obtained from Φ_|*δΩ*_:

(3) $$\begin{array}{c} F\left(\mu_{a}\left(r\right),\kappa \left(r\right)\right)=M. \end{array}$$

The forward model is solved [3] over the domain (V) to estimate the flux density (Φ^predicted^ = *ℳ*[Φ]) on the surface boundary (*Ω*). Due to spatial (*r* ∈ *V*, *Ω*) variation of optical parameter (Δ*μ* ^*i*^(*r*)), the perturbation equation in terms of optical parameter can be written in Taylor series expansion, retaining only first derivative as

(4) ΔMi=Φmeasuredcal−Φpredicted(i)=F′(μi)[Δμi],μi+1(r)=μi(r)+Δμi(r),

where (*F*′) is Jacobian matrix [4, 6] of forward operator *F*.

The image reconstruction problem seeks to find a solution (*μ*_*a*_, *κ*(*r*)) such that the difference between the model predicted *F*(*μ*_*a*_, *κ*) and the experimental measurement (*M* ^*E*^) is minimum. To minimize the error, the cost functional *χ*(*μ*_*a*_, *κ*) is minimized and the cost functional is defined as [4]

(5) $$\begin{array}{c} \chi \left(\mu_{a},k\right)=\arg\left\{\text{mi}{\text{n}}_{\mu_{a},k}||\left[M^{E}-F\left(\mu_{a},k\right)\right]||\right\}, \end{array}$$

where ||·|| is *L*_2_ norm. Through Gauss-Newton and Levenberg-Marquardt [36, 37] algorithms, the minimized form of the above equation for the optical parameter update can be written as

(6) $$\begin{array}{c} \Delta \mu ={\left[J^{T}J+\lambda I\right]}^{-1}J^{T}\Delta M. \end{array}$$

The unknown parameter vectors are recovered from partial and noisy boundary data. This calls for a regularization to yield meaningful results.

### 2.2. Broyden Based MoBIIR

Newton's method is the most popular approach amongst DOT researchers [1, 3–5, 14]. However, because of the repeated evaluation of Jacobian, the high computational complexity of the Newton method has been a major constraint [38–40]. It has been found that the evaluation of Jacobian takes almost 60% of the computation time. Biswas et al. [23, 41] have proposed an algorithm based on Broyden's approach [38] that is found to reduce the computation cost of Jacobian update by an order of magnitude.

Broyden method uses the current estimate of the Jacobian *J*_*i*−1_ and improves it by taking the solution of the secant equation that is a minimal modification to *J*_*i*−1_ (minimal in the sense of minimizing the Frobenius norm ||*J*_*i*_−*J*_*i*−1_||_Frob_). The update is a rank-one update.

The Broyden Jacobian update equation is given as [23]

(7) Ji+1=Ji+[ΔMi−JiΔμi]ΔμiT[Δμi·Δμi]Ji+1=Ji+ΔJi where  ΔJi=[ΔMi−JiΔμi]ΔμiT[Δμi·Δμi].

Equation (2.2) is referred to as Broyden's update equation. The initial value of the Jacobian [*J*(*μ* ^0^)] is computed through analytical method. Since Broyden's method avoids direct computation of Jacobian, this approach provides a computationally simple algorithm [23, 41].

## 3. Discretization of the Diffusion Equation Using Finite Element Method (FEM)

In the forward equation (1), one seeks an approximate solution of the photon density distribution *ϕ*(*r*, *t*). The forward light transport equation DE is discretized using FEM. One of the simplest approximations of *ϕ*(*r*, *t*) is a continuous piecewise linear function $\phi (\hat{r},t)$ which is a linear combination of finite number of piecewise linear basis functions *b*_*i*_(*r*); that is, $\phi (\hat{r},t)=\sum_{i=1}^{N}\phi_{i}(t)b_{i}(r)$. The domain *V* over which the function *ϕ*(*r*, *t*) is defined is divided into a finite set of disjoint elements. The basis functions *b*_*i*_(*r*) have only limited support, limited to a particular element, and are in turn made up of shape functions which are piecewise linear.

In the so-called Galerkin method [2, 4, 5, 17], a weak solution of the DE is sought by requiring that the inner product of the sum of the residuals *R*_*l*_ over all nodal points with the same basis functions vanishes over *V*. Here the residual is

(8) ∑i∑j∫V[1c∂∂t−∇·k(r)∇+μa(r)]ϕi(t)bi(r)−q0(r,t)bj(r)dV=0.

In other words, we require

(9) $$\begin{array}{c} \underset{i}{\sum }\underset{j}{\sum }\int_{V}R_{l}\left(r,t\right)b_{j}\left(r\right)dV=0. \end{array}$$

**Figure 1:**
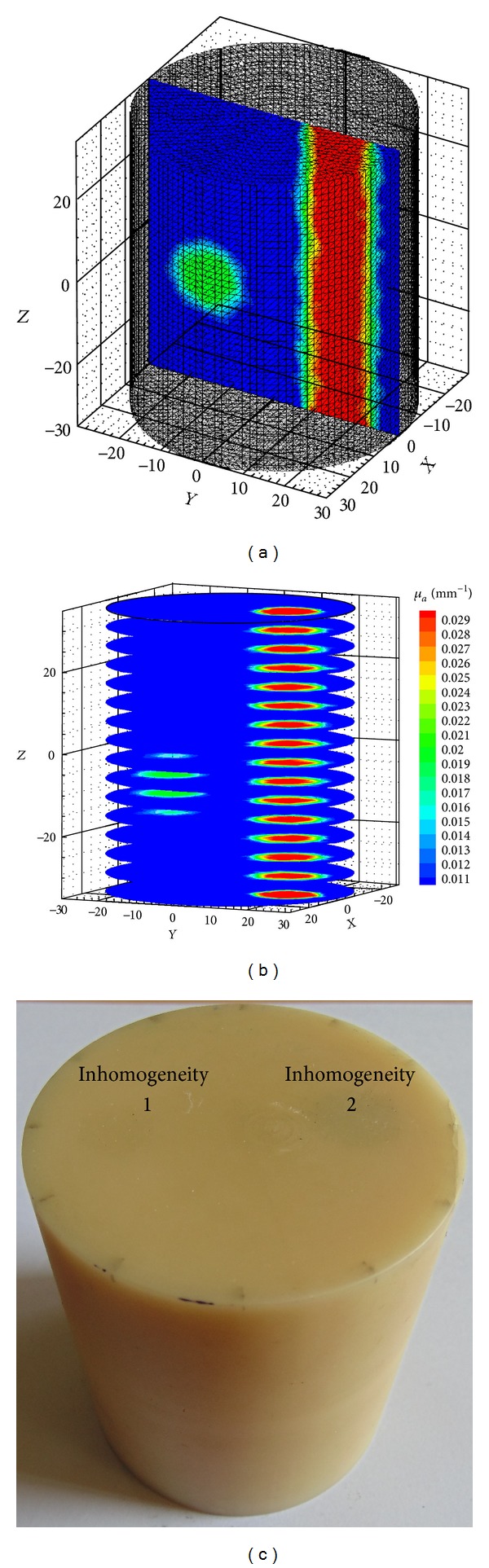
(a) The reference phantom with two inhomogeneities (*μ*_*a*_ = 0.02 mm^−1^ and *μ*_*a*_  = 0.03 mm^−1^), (b) planes along the *Z*-direction, and (c) experimental phantom with two inhomogeneities.

Since the basis functions have only local support limited to individual elements the integrals appearing in the above equation can be split element-wise and evaluated [2]. The amount of data required to establish the computational domain and boundary conditions becomes significantly greater in three-dimensional than two-dimensional problems. We discretized the 3D cylindrical object of 60 mm diameter and 70 mm height (Figure 1) into 66514 tetrahedral elements and 15031 nodes.

To find the properties of the overall system, we must combine the matrix equations of each tetrahedral element in such a way that the resulting matrix represents the behavior of the entire solution region of the problem. The boundary conditions must be incorporated after the assemblage of the individual element contributions.

The discretized, weak form of (1) is evaluated:

(10) $$\begin{array}{c} \left[K\left(k\right)+C\left(\mu_{a}\right)+F\right]=Q. \end{array}$$

Here *K*, *C*, and *F* are sparse matrices whose elements are given by (8). *K* is the global stiffness matrix, which is the assemblage of the individual contribution from diffusion coefficient *κ*(*r*) and absorption coefficient *μ*_*a*_(*r*). *K* has a dimension *N*_*n*_ × *N*_*n*_, where *N*_*n*_ is the number of nodes and is highly sparse, generally with a banded structure. *Q* is the forcing term.

In this study, we used a 3D cylindrical phantom of height 70 mm and diameter 60 mm for our simulation studies and a tissue mimicking phantom having size and parameters matching that of the simulation phantom for experimental validation of the algorithms. The reference phantom is shown in Figure 1(a). 3D cylindrical meshes consisting of 14610, 30823, and 66514 tetrahedral elements have been used for modelling. The background absorption and scattering coefficients are *μ*_*a*_ = 0.01 mm^−1^ and *μ*_*s*_′ = 1.0 mm^−1^, respectively. Two absorbing inhomogeneities of different geometries and contrasts were embedded inside the homogeneous phantom (Figures 1(a) and 1(b)). One inhomogeneity is spherical and has a diameter of 7.9 mm, centered at (0, −16, −10) and the other is cylinder of diameter 7.9 mm, height 70 mm and parallel to *z*-axis. The absorption coefficients of the two inhomogeneities are 0.02 mm^−1^ and 0.03 mm^−1^, respectively. In the experimental phantom, we embedded two cylindrical inhomogeneities of sizes 10 and 12 mm running the whole length of the phantom. The absorption coefficients were 0.02 and 0.035 mm^−1^, respectively.

## 4. Experimental System

**Figure 2:**
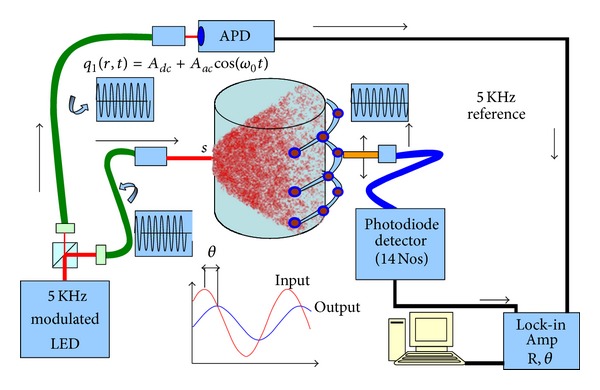
System setup.

For validating our simulation results using GPU with experimental measurement data, we designed and developed a fully 3D DOT system based on frequency domain approach (Figure 2). The source is an intensity modulated led (Thorlabs Mounted LED 850L2 driven by Thorlabs current driver LEDD 1B) emitting light at 850 nm. The led is modulated by 5 KHz sinusoidal current of 20 mA superimposed onto a 100 mA DC current. The output from the led is split using 10 : 1 beam-splitter. The smaller component is connected to an avalanche photodiode (APD) and this forms the reference signal for the lock-in amplifier. The intense part of the light from the beam splitter is coupled to a multimode fibre which delivers light to the cylindrical phantom. A lens which is transparent at the NIR region at the end of the fibre renders the output beam parallel at the phantom surface. The modulated light propagates through the phantom and exits from the boundary. The exiting light is collected at the opposite side by a fibre bundle (diameter 5 mm), which carries light to a photodetector (DET36A from Thorlabs), output of which is connected to a lock-in amplifier. The lock-in amplifier gives the modulation depth and phase shift of photon flux. The schematic diagram of the experimental setup is shown in Figure 2. In order to make a simple and fast system, we used only one source and 14/21 detectors which spans two/three measurement planes. The source and the detector are moved around the phantom using stepper motors. The measurements are taken for 12 source positions and 7 detector positions for each plane. We have carried out the measurements for 2 and 3 planes.

## 5. GPU-Accelerated DOT on CUDA Platform

### 5.1. Assemble the System Matrix

First we assemble the elemental stiffness matrix which comprises 4 nodes of a tetrahedron. The 4 × 4  *k* elemental matrix so formed for each element has to be merged into the global system matrix *K* based on global node numbers. For *n*th element, the equation will be *K*_*n*_ *u*_*n*_ = *f*_*n*_. The entire set of assembled FEM equations is the global stiffness matrix *K*  for the system.

The global system matrix *K* is formed from 66514 elemental *k* matrices. The elemental *k* matrix is estimated using the following approach:

1. coordinates of the 4 nodes of the tetrahedron element *n* to form 4 × 4 coordinate matrix,
2. compute the determinant to arrive at the volume of element *n*,
3. estimate elemental *k* matrix using coordinates of nodes of the element,
4. place the elemental matrix at appropriate place in global *K* matrix.

**Figure 3:**
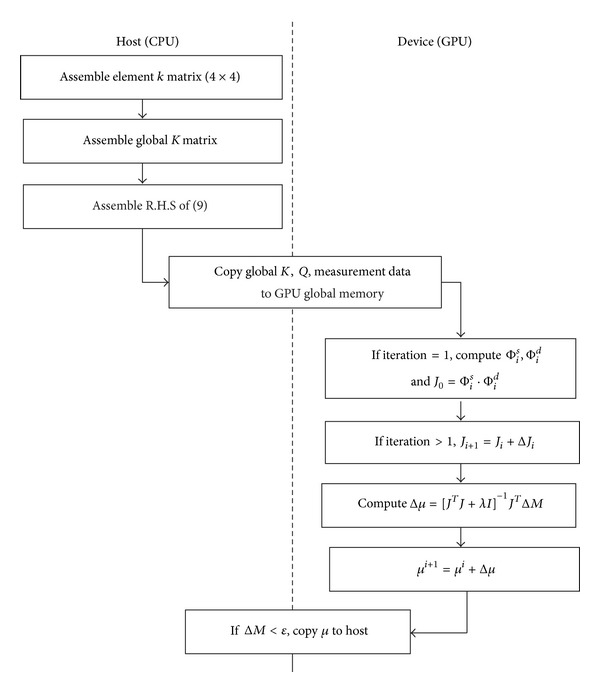
Data flow between the host CPU and GPU device.

The data flow between host and GPU for the solution of the DOT problem is shown in Figure 3. The setting up of global *K* matrix from constituent 66514 tetrahedral elements and the source term *Q* in (10) are carried out in host CPU and then transferred to the GPU memory. The GPU computes the following.

1. It computes *ϕ*_*s*_ and *ϕ*_*d*_ to estimate Jacobian *J* using conventional approach (*J* = *ϕ*_*s*_ · *ϕ*_*d*_). *ϕ*_*s*_ is solved using *ϕ*_*s*_ = *K* ^−1^ · *Q*.
2. The equation (*J* ^*T*^ · *J* + *λ* · *I*)Δ*μ* = *J* ^*T*^ · Δ*M* is assembled and solved for Δ*μ*.
3. *μ*_*i*+1_ is updated with *μ*_*i*_ + Δ*μ*_*i*_.

The solution *μ* is copied back to host memory and host computes termination conditions. If iterations have to be continued, GPU carries it out, because all the data required are already in the GPU. Final results are copied to the host.

### 5.2. GPU C Implementation

The host CPU and GPU specifications of the system we used for evaluating the performance of the algorithms are listed in Table 1. In CUDA architecture, host CPU is the host processor and GPU is the coprocessor. We implement heterogeneous computing concept to offload compute-intensive tasks from the host CPU to the GPU. The single source code comprises standard C host code and device code written using ANSI C extended with keywords for data-parallel functions, called kernels, and their associated data structures. Execution of a CUDA kernel invokes multiple threads which is the basic execution unit organized into thread blocks on a grid. CUDA is capable of running thousands of such inexpensive threads concurrently. The CULA library is GPU-accelerated LAPACK routines which takes advantages of massively parallel NVIDIA CUDA computing architecture to speed up linear algebra.

### 5.3. GPU-Accelerated MATLAB Implementation

MATLAB [25] is a high performance numerical computing environment that uses highly efficient libraries such as ATLAS, LAPACK, and BLAS for numerical linear algebra algorithms. Employing GPU as a coprocessor for these algorithms can accelerate code execution tremendously. MATLAB's Parallel Computing Toolbox provides GPU programming support to take advantage of GPUs from within high-level programming environments. Instead of writing, optimizing, and tuning C or Fortran code for GPUs we can execute preexisting MATLAB code on GPU with minor modifications in the code. MATLAB Parallel Computing Toolbox is providing special array type GPUArray and more than 100 built-in functions that can be directly executed in GPU. We transfer data from the host MATLAB workspace to GPU global memory by* gpuArray* command and run the function on the data in the GPU. The result can be kept in GPU for further operations or can be retrieved from GPU to the host MATLAB workspace as a regular MATLAB variable by using the* gather* command. We first developed MATLAB code for the 3D DOT image reconstruction. The profiling tool presents the execution time of each of the functions. This allows us to identify the tasks that need optimization or GPU execution for speedup. The data movement across the host and GPU is time-consuming. We avoided repeated data exchange between the host CPU and GPU by executing as much computation as possible on GPU using the data transferred to GPU.

We developed 4 types of codes for Broyden based DOT reconstruction algorithm. They are

1. C serial code to execute in host CPU (C CPU),
2. C CUDA code to utilize GPU (C GPU),
3. MATLAB serial code to execute in host CPU (MATLAB CPU),
4. MATLAB single/double precession GPU code (MATLAB GPU).

## 6. Results and Discussion

**Figure 4:**
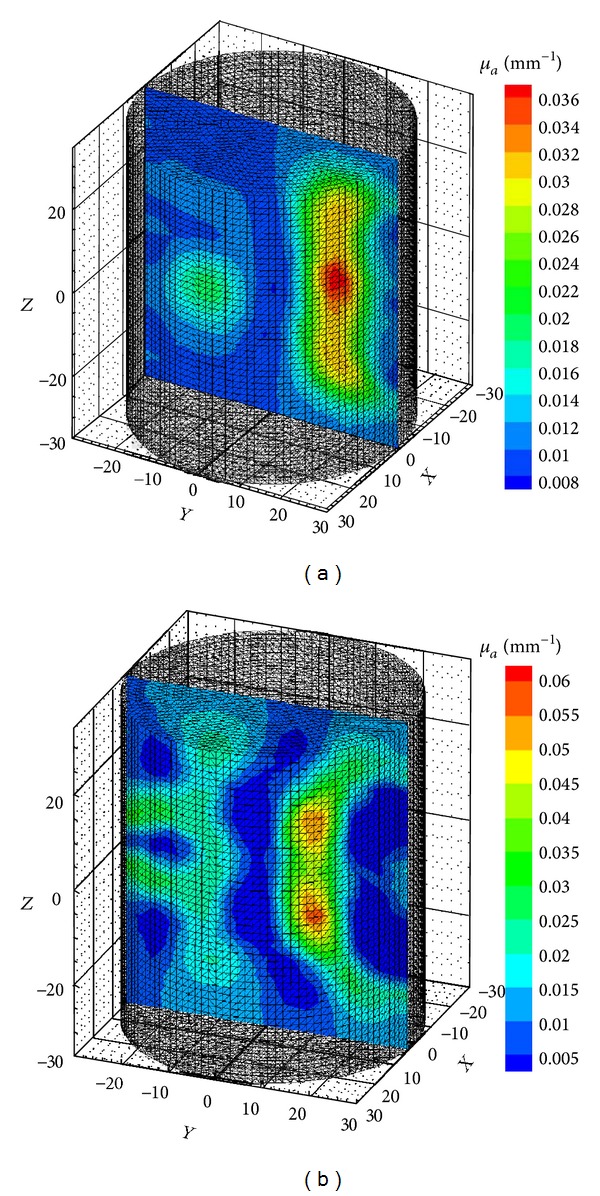
Reconstructed 3D images using C GPU C code (a) for simulation phantom. The phantom has two inhomogeneities, one cylindrical and the other spherical having *μ*_*a*_ of 0.03 and 0.02 mm^−1^, respectively, and (b) experimental phantom with two cylindrical inhomogeneities having diameters of 10 and 12 mm and absorption coefficients of 0.02 and 0.035 mm^−1^, respectively. The background absorption and scattering coefficients are 0.005 mm^−1^and 0.8 mm^−1^. The inhomogeneities run through the length of the phantom.

**Figure 5:**
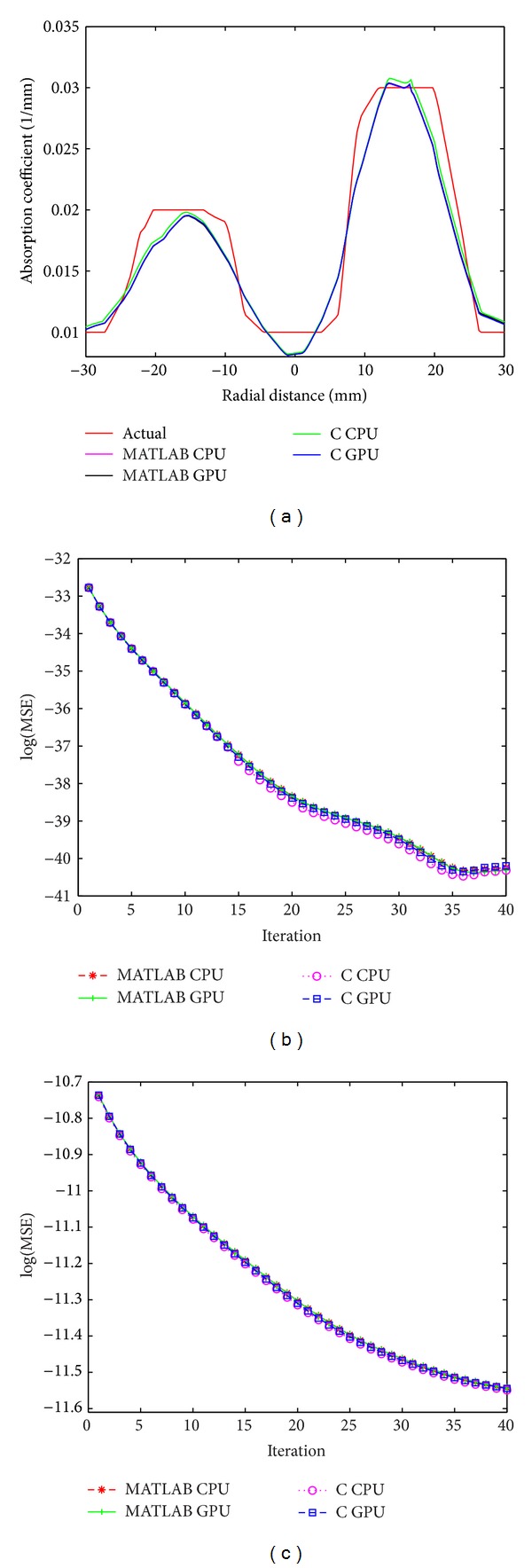
For simulation phantom, (a) cross-sectional line through the center of inhomogeneities 1 and 2, (b) MSE between computed measurements and the experimental measurement data as iteration proceeds (*M* ^*C*^ and *M* ^*E*^), and (c) MSE between *μ*_actual_ and *μ* ^*i*^.

We reconstructed 3D images of the simulation and experimental phantoms (Figure 1) using both C and MATLAB approaches. The reconstructed images using these approaches for highest value of the mesh size for the phantoms are shown in Figure 4.  Figure 4(a) shows the reconstructed image for the simulation phantom, and Figure 4(b) is the result for experimental phantom. The reconstruction quality and contrast are evaluated by using (a) the line plot of the cross-section of the images through the inhomogeneities and (b) the mean square error (MSE) plot. The square of the mean difference (MSE) between the predicted measurement and the actual measurements and the difference between the actual *μ*_*a*_^actual^ and the estimated *μ*_*a*_ are shown in Figures 5(b) and 5(c), respectively. The cross-sectional line plot through the center of two inhomogeneities of the reconstructed images of simulation phantom is given in Figure 5(a). The corresponding results for the experimental phantom are given in Figures 7(a) and 7(b). The cross-sectional plot shows that the localization, contrasts, and the sizes of the inhomogeneities in the reconstruction results are good.

**Figure 6:**
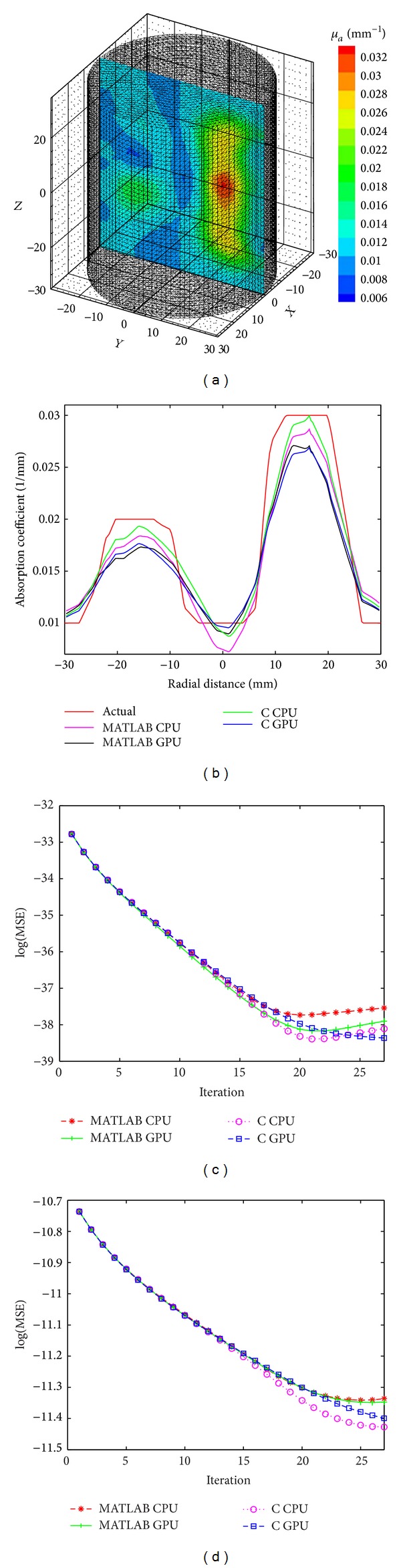
(a) Result of reconstruction of 3D images from 2% noisy data for simulation phantom, (b) cross-sectional line through the center of inhomogeneities 1 and 2, (c) MSE between *M* ^*C*^ and *M* ^*E*^, and (d) MSE between *μ*_actual_ and *μ* ^*i*^.

**Figure 7:**
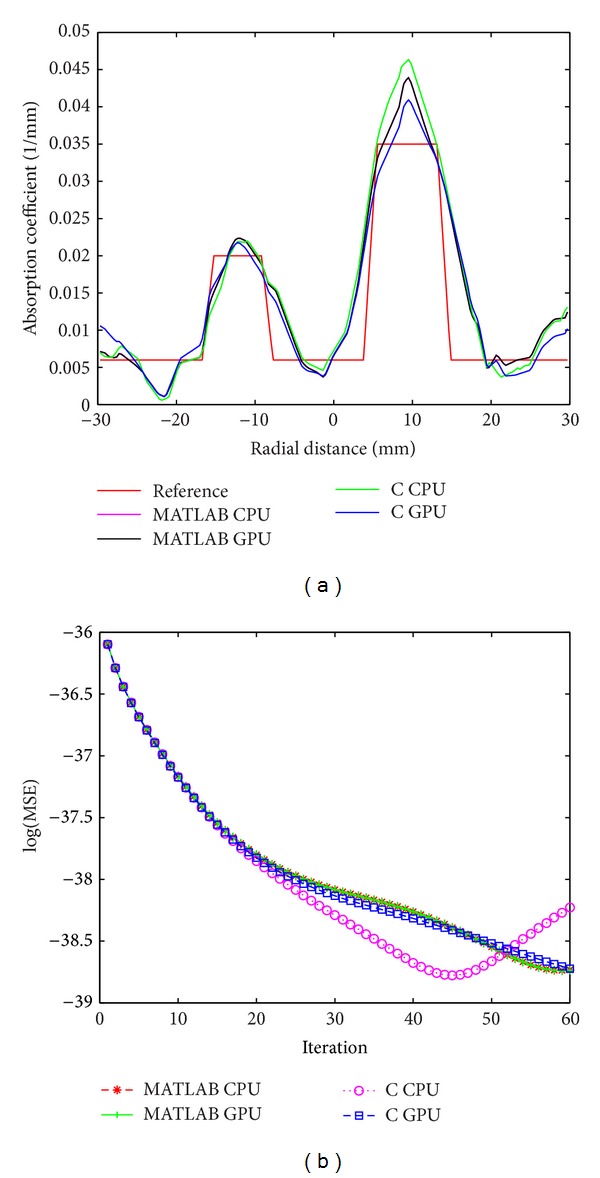
For experimental phantom, (a) cross-sectional line through the center of inhomogeneities 1 and 2 and (b) MSE between computed measurements and the experimental measurement data as iteration proceeds (*M* ^*C*^ and *M* ^*E*^).

In order to verify the stability of the algorithm, we have reconstructed images from noisy measurement data. White Gaussian noise (WGN) of 2% was added to the measurement data [42]. The cross-sectional line plots and MSEs of the reconstruction results are shown in Figures 6(a)–6(c).

**Figure 8:**
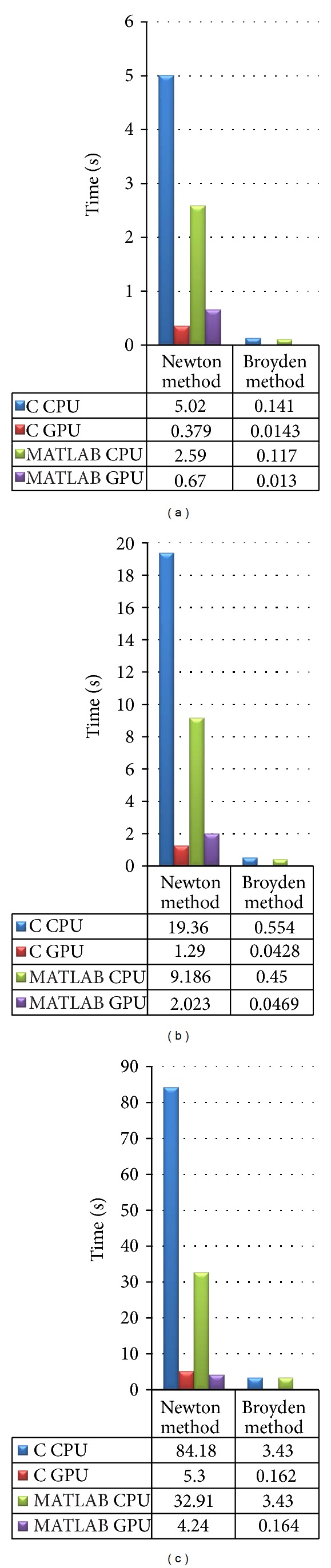
Execution time for Jacobian calculation by conventional and Broyden method on C CPU, C GPU, MATLAB CPU, and MATLAB GPU platforms for mesh size (a) 3736, (b) 7465, and (c) 15031.

**Figure 9:**
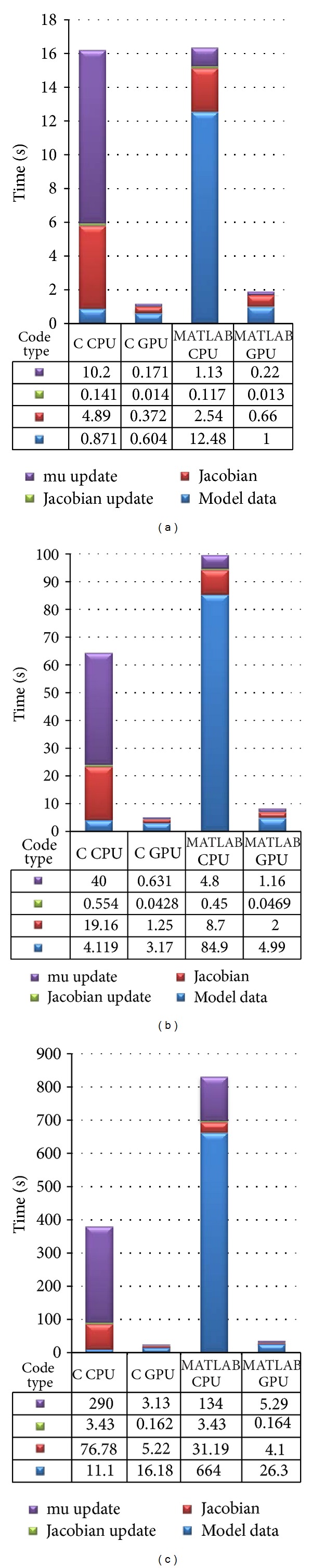
Breakup of the execution time of various computing blocks of the algorithm on C CPU, C GPU, MATLAB CPU, and MATLAB GPU platforms for mesh size (a) 3736, (b) 7465, and (c) 15031.

**Figure 10:**
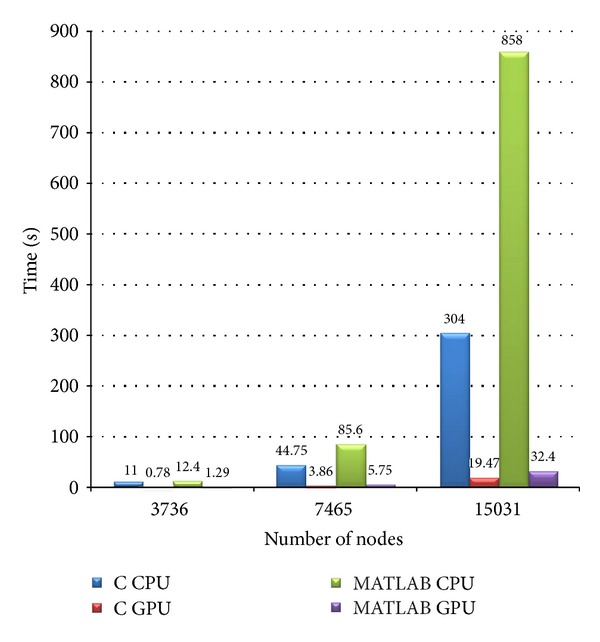
Execution time for an iteration on C CPU, C GPU, MATLAB CPU, and MATLAB GPU platforms for different mesh sizes.

The main focus of the study is to evaluate the performance of the GPU implementations (both C and MATLAB). The Jacobian computation time for conventional and Broyden based methods on C CPU, C GPU, MATLAB CPU, and MATLAB GPU platforms for mesh sizes (a) 14610, (b) 30823, and (c) 66514 elements is presented in Figures 8(a)–8(c), respectively. The breakup of the execution time of various computational blocks of the algorithm on C CPU, C GPU, MATLAB CPU, and MATLAB GPU platforms is shown in Figures 9(a)–9(c), respectively. The plot gives us a picture of the parallelization efficiency of various computational blocks of the algorithm. The execution time for an iteration on C CPU, C GPU, MATLAB CPU, and MATLAB GPU platforms for different mesh sizes is shown in Figure 10. The communication overhead (data transfer from host to GPU and back) of the GPU system is minimized by getting most of the computational tasks executed on GPU, with minimal data movement across the processors.

The memory size available on GPU board (5 GB) puts an upper limit on the mesh size that can be implemented. The current study uses only full matrix computation. For a higher mesh size, we need to resort to sparse matrix computations that will ease the memory requirements. A few studies have been reported using sparse matrix computation because of the sheer size of the problem [31, 35]. Freiberger et al. [35] used an optimized storage approach for sparse matrix elements. It was based on blocked interleaved compressed row storage. The interleaving approach allowed efficient access of the row elements by the GPU threads. The solution of the diffusion equation heavily depended on sparse matrix vector products. Prakash et al. [31] used CUSP package that is capable of dealing with sparse system solver for the real type allowing matrix vector computations. They have shown that even with the use of the full (nonsparse) matrices, the GPUs are capable of giving an acceleration of up to 7 (for completing a start-to-end single iteration of the diffuse optical image reconstruction) compared to CPU sparse computations. We used the conventional sparse matrix storage format CSR (compressed sparse row) for our implementation. The system matrix in sparse matrix format was sent to GPU, and GPU does execute a sparse system solver. However, the current version of CULA does not support the access of these resulting elements in compressed format from the host CPU. The benefit in terms of compute time is lost during the to-and-fro transfer. The next version of CULA will have this option available and that will speed up the execution time still further.

The development of MATLAB based GPU application opens up large opportunities. It has many advantages which include (1) faster development of applications because MATLAB has a rich collection of libraries and functions and (2) much easier development of application program in MATLAB compared to that using conventional C based programming language.

## 7. Conclusions

We have developed an efficient, GPU-based fully 3D tomographic system for diffuse optical tomography (DOT). The 3D DOT, a severely ill-posed and ill-conditioned problem, is solved by making use of the recently proposed Broyden approach for updating the Jacobian matrix, which has computational complexity orders of magnitude lower compared to conventional Jacobian update strategy. The GPU acceleration of the algorithm resulted in a reconstruction speed of 2 frames/second.

## Figures and Tables

**Table 1:** Test systems.

Processing unit	Number of cores	Memory (GB)	Stream multiprocessor (SM)
CPU AMD 8150 @ 3.6 GHz	8	16	
NVIDIA Tesla K20C @ 0.7 GHz	2496	5	13
